# Supplementary material for: New fossil remains of Homo naledi from the Lesedi Chamber, South Africa
Source: eLife. 2017 May 9;6:e24232. doi: 10.7554/eLife.24232 (PMC5423776; doi:10.7554/eLife.24232)
Supplement: Supplementary file 1. — DOI: http://dx.doi.org/10.7554/eLife.24232.045 [file elife-24232-supp1.docx]

**Supplementary file 1. Traits of LES1 cranium in comparison to *H. naledi* and other hominin species.** The samples representing each of these species are those described in the diagnosis of *H. naledi* (Berger et al. 2015), and the character states for all species are as reported in that prior publication. This table adds LES1, which shares nearly all nonmetric characters with *H. naledi*, or where *H. naledi* is variable, occurs within the range of *H. naledi*. A subset of these characters exhibiting variation among species of *Homo* and *Au. sediba* have been selected for Figure 34 in the main text.

| **Cranial Characters** | Cranial capacity | Cranial vault thickness | Supraorbital height index | Postorbital constriction | Frontal bossing | Parietal bossing | Metopic keeling | Sagittal keeling | Parietal wall verticality | Postbregmatic depression |
| --- | --- | --- | --- | --- | --- | --- | --- | --- | --- | --- |
| ***G. gorilla*** | Small | Thin | Low | Marked | Absent | Absent | Absent | Present | Tapered | Present |
| ***P. troglodytes*** | Small | Thin | Low | Moderate | Absent | Absent | Absent & Present | Present | Tapered | Present |
| ***Au. afarensis*** | Small | Thin & Intermediate & Thick | Low | Moderate | Absent | Present | Absent | Absent | Tapered | Absent |
| ***Au. africanus*** | Small | Thin | High | Moderate | Absent | Absent | Absent | Absent | Tapered | Present |
| ***Au. sediba*** | Small | Thin | High | Slight | Present | Present | Absent | Absent | Vertical | Present |
| ***H. naledi*** | Small & Intermediate | Thin | High | Slight | Present | Present | Absent | Present | Vertical | Present |
| **LES1** | Intermediate | Thin | High | Slight | Present | Present | Absent | Absent | Vertical | Present |
| ***H. habilis*** | Intermediate | Thin | High | Moderate | Present | Present | Absent & Present | Absent | Vertical | Present |
| ***H. rudolfensis*** | Large | Thin | High | Moderate | Absent | Present | Absent | Absent | Vertical | Absent |
| ***H. erectus*** | Intermediate & Large & Very large | Thin & Intermediate & Thick | Low & High | Moderate & Slight | Present | Present | Absent & Present | Absent & Present | Vertical | Present |
| ***H. heidelbergensis*** | Very large | Thin & Intermediate & Thick | Low & High | Slight | Absent & Present | Present | Absent & Present | Absent & Present | Tapered | Absent |
| ***H. neanderthalensis*** | Absolutely large | Thin & Intermediate & Thick | Low & High | Slight | Absent & Present | Absent & Present | Absent & Present | Absent & Present | Vertical | Absent & Present |
| ***H. sapiens*** | Very large & Absolutely large | Thin | High | Slight | Absent & Present | Present | Absent & Present | Absent & Present | Vertical | Absent & Present |
| ***P. aethiopicus*** | Small | Thin | Low | Marked | Absent | Absent | Absent | Present | Tapered | ? |
| ***P. boisei*** | Small | Thin | Low | Marked | Absent | Absent | Absent | Present | Tapered | Present |
| ***P. robustus*** | Small | Thin | Low | Marked | Absent | Absent | Absent | Absent & Present | Tapered | Present |

| **Cranial Characters** | Prelambdoidal flattening | Position of temporal crest on supraorbital torus | AM incursion of temporal lines | Compound temporal /nuchal crest | Temporal squama height | Upper margin of temporal squama | Angular torus | Supramastoid crest development |
| --- | --- | --- | --- | --- | --- | --- | --- | --- |
| ***G. gorilla*** | Present | Superior | Moderate & Strong | Present | Low | Straight | Absent | Weak & Marked |
| ***P. troglodytes*** | Absent | Superior | Weak & Moderate & Strong | Present | Low | Straight | Absent | Marked |
| ***Au. afarensis*** | Absent | Superior | Moderate & Strong | Present | Low & High | Curved | Absent | Marked |
| ***Au. africanus*** | Absent | Superior | Moderate & Strong | Absent | Low | Straight & Curved | Absent | Weak & Marked |
| ***Au. sediba*** | ? | Posterior | Weak | Absent | Low | Straight | Absent | Weak |
| ***H. naledi*** | Present | Posterior | Weak | Absent | Low | Curved | Present | Marked |
| **LES1** | Present |  |  |  |  |  |  |  |
| ***H. habilis*** | Absent | Superior | Weak & Moderate | Absent | Low | Straight | Absent | Marked |
| ***H. rudolfensis*** | Absent | Superior | Weak | Absent | Low | Curved | ? | Marked |
| ***H. erectus*** | Absent & Present | Posterior | Weak | Absent | Low & High | Curved | Absent & Present | Weak & Marked |
| ***H. heidelbergensis*** | Absent & Present | Posterior | Weak | Absent | High | Curved | Absent & Present | Weak |
| ***H. neanderthalensis*** | Absent & Present | Posterior | Weak | Absent | Low & High | Straight & Curved | Absent | Marked |
| ***H. sapiens*** | Absent & Present | Superior | Weak | Absent | High | Curved | Absent | Weak |
| ***P. aethiopicus*** | Absent | Superior | Strong | Present | Low | Curved | Absent | Marked |
| ***P. boisei*** | Present | Superior | Strong | Present | Low & High | Straight & Curved | Absent | Weak |
| ***P. robustus*** | Absent | Superior | Strong | Absent | Low | Curved | Absent | Weak |

| **Cranial Characters** | Point of lateral expansion of root of zygomatic | Angulation of root of zygomatic | Mastoid crest projection | Mastoid/ supramastoid crests | Suprameatal spine | Mandibular fossa depth | AM recess of mandibular fossa | Articular tubercle projection | Postglenoid process size |
| --- | --- | --- | --- | --- | --- | --- | --- | --- | --- |
| ***G. gorilla*** | Above EAM | Angled | Marked | Divergent | Present | Intermediate | Absent | Not or slightly projecting | Large |
| ***P. troglodytes*** | Above EAM & Above mandibular fossa | Angled | Weak & Marked | Divergent | Absent & Present | Shallow | Absent | Not or slightly projecting | Large |
| ***Au. afarensis*** | Above EAM | Parallel | Marked | Divergent | Present | Shallow | Present | Not or slightly projecting | Large |
| ***Au. africanus*** | Above EAM | Parallel | Marked | Divergent | Absent & Present | Intermediate | Present | Not or slightly projecting | Large |
| ***Au. sediba*** | Above mandibular fossa | Parallel | ? | ? | ? | Intermediate | ? | Not or slightly projecting | ? |
| ***H. naledi*** | Above mandibular fossa | Angled | Marked | Divergent & Parallel | Present | Intermediate | Absent | Not or slightly projecting | Small |
| **LES1** | Above mandibular fossa | Angled | Marked | Divergent | Present | Intermediate | Absent | Not or slightly projecting | Small |
| ***H. habilis*** | Above mandibular fossa | Angled | Marked | Divergent | Absent | Intermediate | Present | Not or slightly projecting | Medium & Small |
| ***H. rudolfensis*** | Above mandibular fossa | Angled | ? | ? | Present | Intermediate | ? | Not or slightly projecting | Medium |
| ***H. erectus*** | Above mandibular fossa | Angled | Weak & Marked | Divergent & Parallel | Absent & Present | Intermediate | Absent | Not or slightly projecting & Projecting | Small & Medium |
| ***H. heidelbergensis*** | Above mandibular fossa | Parallel | Marked | Divergent & Parallel | Present | Deep | Present | Not or slightly projecting | Large & Medium |
| ***H. neanderthalensis*** | Above mandibular fossa | Parallel | Weak & Marked | Divergent & Parallel | Present | Shallow | Absent & Present | Not or slightly projecting | Large |
| ***H. sapiens*** | Above mandibular fossa | Parallel | Weak & Marked | Parallel | Absent & Present | Deep | Absent | Not or slightly projecting & Projecting | Small |
| ***P. aethiopicus*** | Above EAM | Parallel | Marked | Divergent | Present | Shallow | Absent | Not or slightly projecting | Medium |
| ***P. boisei*** | Above EAM | Parallel | Marked | Divergent | Absent | Deep | Present | Projecting | Medium & Small |
| ***P. robustus*** | Above EAM | Parallel | Marked | Divergent | Absent | Intermediate | Present | Projecting | Small |

| **Cranial Characters** | Postglenoid and tympanic contact | Entoglenoid process projection | Position of the mandibular fossa relative to temporal squama | Tympanic shape | Vaginal process size | Eustachian process of tympanic | Petrous orientation | External auditory meatus size |
| --- | --- | --- | --- | --- | --- | --- | --- | --- |
| ***G. gorilla*** | No | Projecting | Lateral | Tubular | Absent or small | Present & prominent | Sagittal | Small |
| ***P. troglodytes*** | No | Projecting | Lateral | Tubular | Absent or small | Present & prominent | Sagittal | Small |
| ***Au. afarensis*** | No | Projecting | Lateral | Tubular | Absent or small | Absent or slight | Intermediate | Large |
| ***Au. africanus*** | No | Projecting | Lateral | Plate-like | Absent or small | Present & prominent | Intermediate | Large |
| ***Au. sediba*** | ? | Projecting | Medial | ? | ? | ? | ? | ? |
| ***H. naledi*** | Yes | Projecting | Medial | Plate-like | Absent or small | Present & prominent | Coronal | Small |
| **LES1** | Yes | Projecting | Medial | Plate-like | Absent or small | ? | Coronal | Small |
| ***H. habilis*** | Yes | Projecting | Medial | Plate-like | Absent or small | Absent or slight | Coronal | Large |
| ***H. rudolfensis*** | Yes & No | Projecting | Medial | ? | ? | ? | Coronal | ? |
| ***H. erectus*** | Yes | Projecting | Medial | Plate-like | Moderate to large | Absent or slight | Coronal | Large |
| ***H. heidelbergensis*** | Yes | Not projecting | Medial | Plate-like | Moderate to large | Absent or slight | Coronal | Large |
| ***H. neanderthalensis*** | Yes | Not projecting | Medial | Plate-like | Moderate to large | Absent or slight | Coronal | Large |
| ***H. sapiens*** | Yes | Projecting & Not projecting | Medial | Plate-like | Moderate to large | Absent or slight | Coronal | Large |
| ***P. aethiopicus*** | Yes & No | Projecting | Lateral | Plate-like | Moderate to large | Absent or slight | Coronal | Large |
| ***P. boisei*** | Yes & No | Not projecting | Lateral | Plate-like | Moderate to large | Absent or slight | Coronal | Large |
| ***P. robustus*** | Yes | Projecting | Lateral | Plate-like | Moderate to large | Present & prominent | Coronal | Large |

| **Cranial Characters** | ML position of EAM | Orientation of EAM | Crista petrosa | Mastoid process lateral inflation | Mastoid process size | Fossa for origin of digastric muscle | Juxtamastoid eminence | Occipito-mastoid crest | Asterionic notch |
| --- | --- | --- | --- | --- | --- | --- | --- | --- | --- |
| ***G. gorilla*** | Lateral | Oblique | Absent or weak | Not inflated | Large | Broad, shallow fossa | Absent & Present | Absent & Present | Present |
| ***P. troglodytes*** | Medial | Oblique | Absent or weak | Not inflated | Small | Broad, shallow fossa | Absent & Present | Absent & Present | Present |
| ***Au. afarensis*** | Medial | Vertical | Absent or weak | Inflated | Large | Broad shallow fossa | Present | Present | Present |
| ***Au. africanus*** | Medial | Vertical | Moderate to strong | Not inflated | Small & Large | Broad shallow fossa | Absent & Present | Absent & Present | Absent |
| ***Au. sediba*** | ? | ? | ? | ? | ? | ? | ? | ? | ? |
| ***H. naledi*** | Medial & Lateral | Oblique | Absent or weak | Inflated | Small | Deep, narrow notch | Present | Present | Absent |
| **LES1** | Medial | Oblique | Weak | Inflated | Small | Deep, narrow notch | Present | Present | Absent |
| ***H. habilis*** | Medial & Lateral | Oblique | Moderate to strong | Not inflated & Inflated | Small | Deep, narrow notch | Present | Present | Absent |
| ***H. rudolfensis*** | Medial | ? | Moderate to strong | Not inflated | ? | ? | ? | ? | Absent |
| ***H. erectus*** | Medial | Oblique & Vertical | Moderate to strong | Not inflated | Small & Large | Broad, shallow fossa & Deep, narrow notch | Present | Present | Absent |
| ***H. heidelbergensis*** | Medial | Oblique & Vertical | Moderate to strong | Not inflated | Small & Large | Broad, shallow fossa & Deep, narrow notch | Absent & Present | Absent & Present | Absent |
| ***H. neanderthalensis*** | Medial | Oblique & Vertical | Moderate to strong | Not inflated | Large | Broad, shallow fossa & Deep, narrow notch | Absent & Present | Absent & Present | Absent |
| ***H. sapiens*** | Medial | Oblique & Vertical | Moderate to strong | Not inflated | Large | Deep, narrow notch | Absent & Present | Absent & Present | Absent |
| ***P. aethiopicus*** | Medial | Vertical | Moderate to strong | Inflated | Large | ? | ? | ? | Present |
| ***P. boisei*** | Lateral | Oblique | Moderate to strong | Inflated | Large | Broad, shallow fossa | Absent | Absent | Absent |
| ***P. robustus*** | Lateral | Vertical | Moderate to strong | Inflated | Small & Large | Deep, narrow notch | Present | Present | Absent |

| **Cranial Characters** | Venous sinus pattern | Occipital torus | Curvature of occipital squama | Curvature of nuchal plane | Height of nuchal area relative to FH | External occipital protuberance | Tuberculum linearum | External occipital crest | Occipital torus / supramastoid crest contact |
| --- | --- | --- | --- | --- | --- | --- | --- | --- | --- |
| ***G. gorilla*** | Transverse | Present | Flat | Flat | High above FH | Absent | Absent & Present | Present | Not continuous |
| ***P. troglodytes*** | Transverse | Present | Convex | Convex | High above FH | Absent | Absent & Present | Present | Continuous |
| ***Au. afarensis*** | Marginal | Absent | Flat & Convex | Flat | High above FH | Absent | Present | Present | Continuous |
| ***Au. africanus*** | Transverse | Present | Convex | Flat | Low near FH or below | Absent | Present | Present | Not continuous |
| ***Au. sediba*** | ? | ? | ? | ? | ? | ? | ? | ? | ? |
| ***H. naledi*** | Transverse | Present | Convex | Flat | Low near FH or below | Present | Present | Present | Not continuous |
| **LES1** | Transverse | Present | Convex | ? | Low near FH or below | ? | ? | ? | Not continuous |
| ***H. habilis*** | Transverse | Present | Flat | Flat | Low near FH or below | Present | Present | Present | Not continuous |
| ***H. rudolfensis*** | Transverse | Absent | Convex | ? | Low near FH or below | Absent | Present | Present | Not continuous |
| ***H. erectus*** | Transverse | Present | Convex & Flat | Convex & Flat | Low near FH or below | Absent | Present | Present | Not continuous |
| ***H. heidelbergensis*** | Transverse | Absent & Present | Convex | Convex & Flat | Low near FH or below | Absent & Present | Present | Absent & Present | Not continuous |
| ***H. neanderthalensis*** | Transverse | Present | Convex | Convex | Low near FH or below | Absent | Absent | Absent | Not continuous |
| ***H. sapiens*** | Transverse | Absent & Present | Convex | Convex | Low near FH or below | Absent & Present | Absent & Present | Present | Not continuous |
| ***P. aethiopicus*** | Transverse | Present | Flat | Flat | Low near FH or below | Absent | Absent | Present | Continuous |
| ***P. boisei*** | Marginal | Present | Flat | Flat | Low near FH or below | Absent | Absent | Present | Not continuous |
| ***P. robustus*** | Marginal | Present | Flat | Flat | Low near FH or below | Present | Present | Present | Not continuous |

| **Cranial Characters** | Supraorbital torus/arch thickness | Supraorbital thickness gradient | Supratoral sulcus | Supraorbital torus development | Supraorbital contour arched | Supraorbital corner shape | Canine fossa | Anterior pillars | Incisors project beyond bicanine line | Nasoalveolar clivus curvature |
| --- | --- | --- | --- | --- | --- | --- | --- | --- | --- | --- |
| ***G. gorilla*** | Thick | Medial to lateral | Present | Torus | Weakly | Angled | Present | Absent | Yes | Convex |
| ***P. troglodytes*** | Thick & Intermediate | Medial to lateral & Lateral to medial | Present | Torus | Arched | Angled | Present | Absent | Yes | Convex |
| ***Au. afarensis*** | Intermediate | Medial to lateral | Absent | Intermediate | Weakly | Angled | Present | Absent | Yes | Convex |
| ***Au. africanus*** | Intermediate & Thin | Medial to lateral & Lateral to medial | Absent | Intermediate | Arched | Rounded | Absent & Present | Present | Yes | Straight |
| ***Au. sediba*** | Intermediate | Medial to lateral | Present | Torus | Arched | Angled | Present | Absent | Yes | Convex |
| ***H. naledi*** | Intermediate | Medial to lateral | Present | Torus | Weakly | Rounded | Present | Absent | Yes | Convex |
| **LES1** | Intermediate | Medial to lateral | Present | Torus | Weakly | Rounded | Present | Absent | Yes | Convex |
| ***H. habilis*** | Intermediate | Medial to lateral & Lateral to medial | Present | Torus | Arched | Rounded | Present | Absent & Present | Yes | Straight & Convex |
| ***H. rudolfensis*** | Intermediate | Medial to lateral & Lateral to medial | Absent | Intermediate | Arched | Rounded | Absent | Absent | No | Straight |
| ***H. erectus*** | Thick & Intermediate | Medial to lateral & Lateral to medial | Present | Torus | Weakly& Arched | Angled & Rounded | Present & Absent | Absent | Yes | Convex |
| ***H. heidelbergensis*** | Thick & Intermediate | Medial to lateral | Present & Absent | Intermediate | Arched | Rounded | Absent | Absent | Yes | Convex |
| ***H. neanderthalensis*** | Intermediate | Medial to lateral | Absent | Torus | Arched | Rounded | Absent | Absent | Yes | Convex |
| ***H. sapiens*** | Intermediate & Thin | Medial to lateral | Absent | Weak | Weakly& Arched | Rounded | Present | Absent | Yes | Convex |
| ***P. aethiopicus*** | Intermediate | Lateral to medial | Absent | Torus | Weakly | Rounded | Absent | Absent | No | Concave |
| ***P. boisei*** | Thick | Lateral to medial | Absent | Torus | Arched | Rounded | Absent | Absent | No | Concave |
| ***P. robustus*** | Intermediate | Lateral to medial | Absent | Torus | Arched | Rounded | Present | Present | No | Concave |

| **Cranial Characters** | Nasoalveolar clivus contour | Canine jugum development | Anterior zygomatico-alveolar position | Intermaxillary suture development | Lateral flaring of zygomatic arches | Zygomatic temporal surface | Zygomatic frontal process lateral margin orientation | Anterior palate depth | Maxillo-alveolar relative length | Position of incisive foramen |
| --- | --- | --- | --- | --- | --- | --- | --- | --- | --- | --- |
| ***G. gorilla*** | Convex | Marked | M1M2 | Furrowed | Slight | Shallow | Vertical | Shallow | Longer than wide | Canine |
| ***P. troglodytes*** | Convex | Marked | M1M2&M1 | Furrowed | Slight | Shallow | Vertical | Shallow | Longer than wide | Canine |
| ***Au. afarensis*** | Convex | Marked | P3P4&P4M1 | Furrowed | Marked | Deeply excavated | Laterally divergent | Shallow | Longer than wide & Broader than long | P3 |
| ***Au. africanus*** | Flat or slightly concave | Marked | P4M1&M1 | Furrowed & Flat | Marked | Shallow | Laterally divergent | Shallow & Deep | Longer than wide | P3 |
| ***Au. sediba*** | Convex | Marked | P4M1 | Ridged | Slight | Shallow | Vertical | Deep | Longer than wide | P3 |
| ***H. naledi*** | Flat or slightly concave | Weak | P3P4 | Ridged | Slight | Shallow | Vertical | Shallow | Broader than long | P3 |
| **LES1** | Slightly concave | Weak | P3P4 | Ridged | ? | ? | ? | Shallow | Broader than long | P3 |
| ***H. habilis*** | Convex | Moderate | P4M1&M1 | Ridged | Slight | Shallow | Vertical | Shallow | Broader than long | P3 |
| ***H. rudolfensis*** | Flat or slightly concave | Weak | P4M1 | Flat | Slight | Shallow | Vertical | Deep | Broader than long | P3 |
| ***H. erectus*** | Convex & Flat or slightly concave | Weak & Moderate | P4M1&M1 | Ridged | Slight | Shallow | Vertical | Deep | Broader than long | P3 |
| ***H. heidelbergensis*** | Convex | Weak | P4M1&M1 | Ridged | Slight | Shallow | Vertical | Deep | Broader than long | P3 |
| ***H. neanderthalensis*** | Convex | Weak | M1&M1M2 | Ridged | Slight | Shallow | Vertical | Deep | Broader than long | Canine |
| ***H. sapiens*** | Convex & Flat or slightly concave | Weak | P4M1&M1&M1M2 | Ridged | Slight | Shallow | Vertical | Shallow | Broader than long | Canine |
| ***P. aethiopicus*** | Flat or slightly concave | Weak | P3P4 | Furrowed | Marked | Shallow | Laterally divergent | Shallow | Longer than wide | P3 |
| ***P. boisei*** | Flat or slightly concave | Weak | P3P4 | Furrowed | Marked | Shallow | Laterally divergent | Shallow & Deep | Longer than wide | P3 |
| ***P. robustus*** | Flat or slightly concave | Weak | P3P4 | Furrowed | Marked | Shallow | Laterally divergent | Shallow | Broader than long | P3 |

| **Mandibular Characters** | Mandibular symphysis area | Transverse tori development | Post-incisive planum | Cross-sectional area at M1 | Orientation of mandibular symphysis | Mental foramen opening direction | Hollowing above/behind mental foramen | Mandibular extramolar sulcus width |
| --- | --- | --- | --- | --- | --- | --- | --- | --- |
| ***G. gorilla*** | Small & Large | Sup. torus stronger than inf. | Prominent | Small & Large | Receding | Anterior & Lateral | Present | Wide |
| ***P. troglodytes*** | Small & Large | Inf. torus stronger than sup. | Weak | Small | Receding | Anterior | Present | Wide |
| ***Au. afarensis*** | Small & Large | Inf. torus stronger than sup. | Prominent | Small | Receding | Anterior & Lateral | Present | Wide |
| ***Au. africanus*** | Small | Inf. torus stronger than sup. | Prominent | Small | Receding | Anterior & Lateral | Present & Absent | Wide |
| ***Au. sediba*** | Small | Inf. torus stronger than sup. | Weak | Small | Vertical | Lateral | Present | Wide & Narrow |
| ***H. naledi*** | Small | Inf. torus stronger than sup. | Weak | Small | Vertical | Posterior | Present | Wide |
| **LES1** | Small | Inf. torus stronger than sup. | Weak | Small | Vertical | Posterior | Present | Wide |
| ***H. habilis*** | Small | Sup. torus stronger than inf. & Absent | Prominent | Small | Vertical | Lateral | Absent | Wide |
| ***H. rudolfensis*** | Large | Inf. torus stronger than sup. | Prominent | Small & Large | Vertical | Lateral | Present | Wide |
| ***H. erectus*** | Small | Sup. torus stronger than inf. & Both tori equal & Absent | Prominent | Small | Vertical | Lateral | Present & Absent | Wide |
| ***H. heidelbergensis*** | Small | Inf. torus stronger than sup | Weak | Small | Vertical | Posterior | Absent | Narrow |
| ***H. neanderthalensis*** | Small | Inf. torus stronger than sup | Weak | Small | Vertical | Posterior | Absent | Wide |
| ***H. sapiens*** | Small | Absent | Weak | Small | Vertical | Posterior | Absent | Wide |
| ***P. aethiopicus*** | Small & Large | Inf. torus stronger than sup. | Prominent | Large | Vertical | Lateral | Absent | Wide |
| ***P. boisei*** | Large | Inf. torus stronger than sup. | Prominent & Weak | Large | Vertical | Lateral | Absent | Wide |
| ***P. robustus*** | Large | Inf. torus stronger than sup. | Prominent & Weak | Large | Vertical | Lateral | Absent | Wide |

| **Mandibular Characters** | Orientation of mandibular premolar row (dental arcade shape) | Anterior marginal tubercle position | Mental foramen position | Mental foramen height | Mandibular incisure | Subalveolar fossae |
| --- | --- | --- | --- | --- | --- | --- |
| ***G. gorilla*** | Parasagittal | Absent | P4M1 | Below midcorpus | Absent | Absent or slight |
| ***P. troglodytes*** | Parasagittal | P4M1 | P3P4 | Below midcorpus | Absent | Absent or slight |
| ***Au. afarensis*** | Parasagittal | P3P4 | P3P4 | Below midcorpus | Absent | Moderate to prominent |
| ***Au. africanus*** | Oblique | P3P4 | P3P4 | At midcorpus | Absent | Absent or slight |
| ***Au. sediba*** | Oblique | P3P4 | P3P4 | At midcorpus | Present | Moderate to prominent |
| ***H. naledi*** | Oblique | P3P4 | P3P4 | Above midcorpus | Present | Moderate to prominent |
| **LES1** | Oblique | P3P4 | P3P4 | At midcorpus | Present | Moderate to prominent |
| ***H. habilis*** | Oblique | P3P4 | P3P4 | At midcorpus | Present | Moderate to prominent |
| ***H. rudolfensis*** | Oblique | P3P4 | P3P4 | At midcorpus | Present | Moderate to prominent |
| ***H. erectus*** | Oblique | P3P4 & P4M1 | P3P4 & P4M1 | At midcorpus | Present | Moderate to prominent |
| ***H. heidelbergensis*** | Oblique | M1 | P4M1 & M1 or distal to M1 | Below midcorpus | Absent | Moderate to prominent |
| ***H. neanderthalensis*** | Oblique | Absent & P3P4 & P4M1 & M1 | P4M1 & M1 or distal to M1 | At midcorpus&Above midcorpus | Present | Moderate to prominent |
| ***H. sapiens*** | Oblique | P4M1 | M1 or distal to M1 | Below midcorpus&At midcorpus | Present | Moderate to prominent |
| ***P. aethiopicus*** | Oblique | P4M1 | P3P4 & P4M1 | At midcorpus | Absent | Moderate to prominent |
| ***P. boisei*** | Oblique | P4M1 | P4M1 | Above midcorpus | Absent & Present | Moderate to prominent |
| ***P. robustus*** | Oblique | P3P4 | P3P4 | At midcorpus | Absent | Moderate to prominent |
