# Supplementary material for: New fossil remains of Homo naledi from the Lesedi Chamber, South Africa
Source: eLife. 2017 May 9;6:e24232. doi: 10.7554/eLife.24232 (PMC5423776; doi:10.7554/eLife.24232)
Supplement: Supplementary file 5. — DOI: http://dx.doi.org/10.7554/eLife.24232.049 [file elife-24232-supp5.docx]

**Supplementary file 5. Taphonomic observations by specimen from the Lesedi Chamber.** In this file, first is provided a table of observations, scored in discrete categories (e.g., 0, 1, 2) for each of 19 specimens that represent most of the large bone fragments in the Lesedi hominin sample. Below the data table is included a table that provides a description for each of the taphonomic observations, and details the criteria that were used to score the specimens.

| **Trace** | **Feature** | **LES1**  **man** | **LES1 max** | **102a-001** | **102a-002** | **102a-003** | **102a-004** | **102a-010** | **102a-015** | **102a-020** | **102a-021** | **102a-025** | **102a-036** | **102a-051** | **102a-138** | **102a-206** | **102a-257** | **102a-284** | **102b-438** | **102c-589** |
| --- | --- | --- | --- | --- | --- | --- | --- | --- | --- | --- | --- | --- | --- | --- | --- | --- | --- | --- | --- | --- |
| **Preservation** |  | 2 | 2 | 3 | 2 | 3 | 3 | 2 | 2 | 2 | 1 | 2 | 2 | 3 | 2 | 3 | 2 | 3 | 3 | 3 |
| **Mineral staining** | Iron | 2 | 2 | 2 | 2 | 2 | 2 | 2 | 2 | 2 | 2 | 2 | 0 | 0 | 0 | 2 | 2 | 2 | 2 | 0 |
|  | Stain pattern | 1 | 1 | 1 | 1 | 1 | 1 | 1 | 1 | 1 | 1 | 1 | 0 | 0 | 0 | 1 | 1 | 1 | 1 | 0 |
|  | Manganese (black) | 2 | 1 | 2 | 2 | 2 | 2 | 1 | 2 | 2 | 1 | 1 | 2 | 2 | 2 | 2 | 1 | 2 | 2 | 2 |
|  | Stain pattern | 1 | 1 | 1 | 1, 2 | 1 | 1 | 1, 2 | 1 | 1 | 1, 2 | 1, 2 | 1 | 1 | 1 | 1 | 1, 2 | 1 | 1 | 1 |
|  | Tide mark | 0 | 0 | 0 | 0 | 0 | 0 | 0 | 0 | 0 | 0 | 0 | 0 | 0 | 0 | 0 | 0 | 0 | 0 | 0 |
| **Fracture pattern** | Peri-mortem | 0 | 0 | 2 | 0 | 0 | 0 | 0 | 0 | 0 | 0 | 0 | 0 | 0 | 0 | 0 | 0 | 0 | 0 | 0 |
|  | Post-mortem | 7 | 7 | 0 | 3 | 1 | 2, 3, 6 | 7 | 1 | 1 | 1 | 3 | 0 | 7 | 7 | 1, 2 | 3 | 7 | 0 | 7 |
|  | Crushing | 0 | 0 | 1 | 0 | 1 | 0 | 0 | 0 | 1 | 0 | 0 | 0 | 0 | 0 | 0 | 0 | 0 | 0 | 0 |
|  | Recent fracture/edge wear | 1 | 0 | 0 | 1 | 0 | 1 | 0 | 1 | 1 | 1 | 1 | 0 | 1 | 1 | 0 | 0 | 1 | 1 | 1 |
| **Surface effects** | Cracking | 3 | 4 | 2 | 0 | 2 | 2 | 3 | 2 | 2 | 2 | 2 | 0 | 0 | 0 | 3 | 0 | 0 | 3 | 3 |
|  | Crack penetration | 1 | 1 | 1, 2 | 0 | 1 | 1 | 1 | 1 | 1 | 1 | 1 | 0 | 0 | 0 | 0 | 0 | 0 | 1 | 1 |
|  | Patination (mosaic cracking) | 0 | 0 | 0 | 0 | 0 | 0 | 0 | 0 | 0 | 0 | 0 | 0 | 0 | 0 | 0 | 0 | 0 | 0 | 0 |
|  | Delamination/peeling | 0 | 0 | 0 | 0 | 0 | 0 | 0 | 0 | 0 | 0 | 0 | 0 | 0 | 0 | 0 | 0 | 0 | 0 | 0 |
|  | Bleaching | 0 | 0 | 0 | 0 | 0 | 0 | 0 | 0 | 0 | 0 | 0 | 0 | 0 | 0 | 0 | 0 | 0 | 0 | 0 |
| **Cortical removal** | Type | 2 | 3 | 2 | 3 | 1 | 1 | 1 | 0 | 0 | 1 | 2 | 0 | 0 | 3 | 0 | 0 | 0 | 0 | 0 |
|  | Striations | 0 | 0 | 3 | 1 | 2 | 3 | 1 | 1 | 1 | 0 | 3 | 0 | 0 | 0 | 0 | 1 | 0 | 0 | 0 |
|  | Pitting | 1 | 1 | 1 | 2 | 1 | 1 | 2 | 2 | 1 | 2 | 2 | 1 | 0 | 1 | 1 | 1 | 1 | 0 | 1 |
|  | Pit position | 1 | 1 | 1 | 1 | 1 | 1 | 1 | 1 | 1 | 1 | 1 | 1 | 0 | 1 | 1 | 1 | 1 | 0 | 1 |
|  | Furrow or gouge | 0 | 0 | F, G, 1 | 0 | F, 1 | F, G, 1 | 0 | G, 1 | F, G, 1 | 0 | 0 | 0 | 0 | 0 | 0 | 0 | 0 | 0 | 0 |
| **Modification of mineral deposit** | Penetrates existing mineral surface | 1 | 1 | 1 | 1 | 1 | 1 | 1 | 1 | 0 | 1 | 1 | 0 | 0 | 0 | 1 | 1 | 0 | 0 | 0 |
| **Destruction** | (E) epiphyseal; (N) non-epiphyseal | 0 | N, 1 | E, N, 2 | 0 | E, N, 2 | 0 | 0 | E, N, 2 | 0 | E, 2 | 0 | E, N, 2 | E, N, 2 | N, 1 | 0 | E, 2 | N, 2 | 0 | 0 |
|  | Coffin wear | 0 | 0 | 1 | 0 | 1 | 0 | 0 | 1 | 0 | 0 | 0 | 0 | 0 | 0 | 0 | 0 | 0 | 0 | 0 |
| **Fluvial markers** | (T) thinning; (S) smoothing; (P) polish; (F) frosting; (W) window or aperture | 0 | 0 | 0 | 0 | 0 | 0 | 0 | 0 | 0 | 0 | 0 | 0 | 0 | 0 | 0 | 0 | 0 | W, 1 | 0 |
| **Bore hole** |  | 0 | 0 | 0 | 0 | 0 | 0 | 0 | 0 | 0 | 0 | 0 | 0 | 0 | 0 | 0 | 0 | 0 | 0 | 0 |
| **Cut or chop mark** | (C) cut marks; (P) peeling or shaved defects; (N) point insertions or notched defects; (S) slot fractures; (M) chop marks or scoop defects | 0 | 0 | 0 | 0 | 0 | 0 | 0 | 0 | 0 | 0 | 0 | 0 | 0 | 0 | 0 | 0 | 0 | 0 | 0 |
| **Carnivore modification** | (B) bone cylinders; (P) tooth pits; (S) tooth scores; (E) end scalloping; (G) gastric corrosion | 0 | 0 | 0 | 0 | 0 | 0 | 0 | 0 | 0 | 0 | 0 | 0 | 0 | 0 | 0 | 0 | 0 | 0 | 0 |
| **Rodent** |  | 0 | 0 | 0 | 0 | 0 | 0 | 0 | 0 | 0 | 0 | 0 | 0 | 0 | 0 | 0 | 0 | 0 | 0 | 0 |
| **Burnt** |  | 0 | 0 | 0 | 0 | 0 | 0 | 0 | 0 | 0 | 0 | 0 | 0 | 0 | 0 | 0 | 0 | 0 | 0 | 0 |

**Criteria used for scoring specimens by observation:**

| **Taphonomic character** | **Descriptive criteria** |
| --- | --- |
| **General preservation** | This is an assessment of general bone quality and preservation, encoding information related to the degree of surface modification, surface erosion and cortical integrity. Criteria are modified from *Standards for Recording Human Remains* for skeletal inventories [1]. Four grades defined; (1) denotes slight to patchy surface erosion or modification; (2) more extensive surface erosion than grade 1 with deeper surface penetration; (3) most of bone surface affected by some degree of erosion - general morphology maintained but detail of parts of surface masked by erosive action; (4) majority of bone surface affected by erosive action - general profile maintained and depth of modification not uniform across whole surface. |
| **Mineral staining** | Iron (red) staining. Denotes the presence of iron oxide staining on bone surfaces. Graded as (1) heavy indicating surface staining of more than 50% of the bone surface (may be continuous or discontinuous), or (2) patchy indicating discontinuous coverage over less than 50% of the surface. |
|  | Manganese (black). Denotes the presence of manganese oxyhydroxide staining on bone surfaces. Graded as (1) heavy indicating surface staining of more than 50% of the bone surface (may be continuous or discontinuous), or (2) patchy indicating discontinuous coverage over less than 50% of the surface. |
|  | Stain pattern (of mineral). Indicates the general pattern of mineral staining or deposition on the surface of bone. Recording as: (1) spotted or diffuse patches (may be present as irregular or random spots comprising multiple patches a few millimetres in diameter, or more focussed and slightly larger ‘leopard’ spotting ); (2) a mat or continuous surface of mineral with a surface coverage generally greater than 400 mm^2^. |
|  | Tide mark. This indicates the presence of a longitudinal mineral stain. Stains are visible as single or multiple linear deposits of manganese and/or iron oxy-hydroxide phases, and mark a contact boundary between the bone surface and surrounding sediment, and indicate the resting orientation of the bone during precipitation of the stains [2]. Recorded as (1) present or (0) absent. |
| **Fracture pattern** | Peri-mortem trauma. Identification of fracture patterns consistent with biomechanical markers of green or wet bone failure, using classification criteria from the forensic [3-7] and archaeological literature [8, 9]. Nomenclature based on cross-sectional morphology of fractured ends as presented by Galloway [10-13] consistent with markers of tensile-compressive failure. Fractures are recorded as (1) transverse, (2) spiral, (3) oblique, (4) butterfly, (5) segmental or (6) other (comminuted, longitudinal incomplete, greenstick, torus etc.). |
|  | Post-mortem fractures. Identification of fracture patterns consistent with dry bone breakage using mechanical and gross-morphological classification criteria [6, 8]. Nomenclature based on cross-sectional morphology of fractured ends from Marshall [14] consistent with markers of biomechanically incompetent failure. Fractures are recorded as (1) transverse dry, (2) step or columnar, (3) oblique dry, (4) y-shaped, (5) flaked, (6) longitudinal, (7) other. |
|  | Crushing. Evidence of localised surface compression with retention of comminuted fragments. Recorded as (1) present or (0) absent. |
|  | Recent fracture or edge wear. Evidence of recent damage or abrasion to dry bone as evidenced by mismatch between internal structures or cortex at fresh break points being differentiated by showing as pale buff to off-white in cross-section. Recorded as (1) present or (0) absent. |
| **Surface effects** | Cracking. The presence of incomplete surface disruptions which are observed in cases of sub-aerial (external) weathering [2, 15-21] and sub-surface burial environments [22, 23]. Cracking is recorded as: (1) transverse, across the long axis of a bone or perpendicular to longitudinal cracks; (2) longitudinally along the primary axis of the bone; (3) following the bone grain; (4) other. |
|  | Crack penetration. The extent of penetration into cortical structure, as observed in plan and cross section. Recorded as: (1) superficial where cracking penetrates less than 25% of the cortex as seen in cross section, or observed in plan; (2) deep, where cracks penetrate more than 25% of the cortex as seen in cross section (may extend through cortex as split lines). |
|  | Patination (mosaic cracking). Superficial cracking or crazing across an area greater than 1cm^2^. Recorded as (1) present or (0) absent. |
|  | Delamination/peeling. The presence of rough homogeneously altered cortical bone, with fibrous texture evidenced. Splinters of bone may be present in adhesion or removed, and areas of exfoliation noted. Recorded as (1) present or (0) absent. |
|  | Bleaching (localised or hemi-surface). Recorded as (1) present or (0) absent. |
| **Cortical removal** | Areas of possible gross invertebrate modification of the available cortical surface, consistent with the criteria of Dirks [24] and Backwell [25]. These can occur as (1) focussed or singular spots of outer cortex, (2) multifocal defects, or (3) diffuse areas of radular damage observed under low magnification. |
|  | Striations. Areas of fine radular damage observed under low magnification, consistent with the criteria of Dirks [24] and Backwell [25]. These can occur as (1) single striae, (2) multiple clusters, or (3) diffuse/random in distribution. |
| **Pitting** | Areas of possible gross invertebrate modification of the available cortical surface, consistent with the criteria of Dirks [24] and Backwell [25]. Pitting up to 2mm in diameter, which occurs as round, ovoid or sub-rectangular defects in plan. Recorded as (1) diffuse or (2) occurring in clusters or multi-focal areas. |
|  | Position of pitting. Recorded as (1) random or diffuse, (2) adjacent to a joint surface, or (3) distal (mid-diaphyseal) to a joint surface or epiphysis. |
| **Furrow or gouge** | Short, parallel, and linear or straight marks that may be perpendicular or transverse to the long axis of the bone. Recorded as (F) furrow or (G) gouge; (1) present or (0) absent. |
| **Modification of mineral deposit** | Pit defects or areas of cortical removal which penetrates pre-existing mineral surface deposits or concretions (iron or manganese). Recorded as (1) yes (0) no. |
| **Destruction (underlying structure exposed)** | Areas of cortical removal consistent with invertebrate modification, sediment abrasion or other mechanisms. Recorded as occurring at (E) epiphyses or the joint surface, or (N) non-epiphyseal (diaphyseal or other cortical surface). Extent recorded as (1) single surface or (2) multiple surfaces affected. |
|  | Coffin wear. The patterned, localized destruction of margins of joint surfaces brought about by contact with a hard substrate during decomposition. Colloquially this is referred to as coffin wear. Areas affected include the posterior portions of several elements, including the occipital portion of the cranium, the vertebral spines/arches, the scapulae, the pelvis, and the limb bones whilst in supine or anatomical position [22]. Recorded as (1) present or (0) absent. |
| **Fluvial transport markers** | The presence of trace criteria characteristic of fluvial transport. These comprise: (T) thinning; (S) smoothing; (P) polish; (F) frosting; and (W) window or aperture formation. Identification criteria derived from forensic [26-28] and palaeontological sources [29-31]. Individual coded criteria recorded as (1) present or (0) absent. |
| **Bore hole** | Deep circular or ovoid defects greater than 2mm in diameter, perpendicular to, or running parallel to the bone surface [25, 32]. Recorded as (1) present or (0) absent. |
| **Cut or chop marks** | The presence of trace criteria characteristic of cutting, hacking or chopping. These comprise: (C) cut marks; (P) peeling or shaved defects; (N) point insertions or notched defects; (S) slot fractures; (M) chop marks or scoop defects [33-38]. Individual coded criteria recorded as (1) present or (0) absent. |
| **Carnivore modification** | The presence of trace criteria characteristic of carnivore modification of bone. These comprise: (B) bone cylinders; (P) tooth pits; (S) tooth scores; (E) end scalloping; (G) gastric corrosion [39, 40]. Individual coded criteria recorded as (1) present or (0) absent. |
| **Rodent** | Localised or widespread multiple striated defects to the cortical surface and exposed bone edges [40]. Recorded as (1) present or (0) absent. |
| **Burnt** | Presence of thermal alteration as evidenced by burn line, charring or calcination [41, 42]. Recorded as (1) present or (0) absent. |

1. McKinley, J.I., *Compiling a skeletal inventory: disarticulated and co-mingled remains*, in *Guidelines to the Standards for Recording Human Remains. British Association for Biological Anthropology and Osteoarchaeology & Institute of Field Archaeologists. Technical Paper No. 7.* , M. Brickley and J.I. McKinley, Editors. 2004. p. 14-17.

2. Junod, C.A. and J.T. Pokines, *Subaerial weathering*, in *Manual of Forensic Taphonomy*, J.T. Pokines and S.A. Symes, Editors. 2014, CRC Press: Boca Raton, FL. p. 287-314.

3. Galloway, A., et al., *The role of the forensic anthropologist in trauma analysis*, in *Broken Bones: Anthropological Analysis of Blunt Force Trauma*, A. Galloway, Editor. 1999, Charles C. Thomas: Springfield, IL. p. 5-31.

4. L’Abbé, E.N., et al., *Evidence of fatal skeletal injuries on Malapa Hominins 1 and 2.* Scientific Reports, 2015. **5**: p. 15120.

5. Symes, S., et al., *Interpreting traumatic injury from bone in medico-legal investigations*, in *A Companion to Forensic Anthropology*, D. Dirkmaat, Editor. 2012, Wiley-Blackwell: London. p. 340-389.

6. Symes, S., et al., *Taphonomy and the timing of bone fractures in trauma analysis*, in *Manual of Forensic Taphonomy*, J. Pokines and S. Symes, Editors. 2014, CRC Press: Boca Raton, FL. p. 341-365.

7. Loe, L., *Perimortem trauma*, in *Handbook of Forensic Anthropology and Archaeology*, S. Blau and D.H. Ubelaker, Editors. 2009, Left Coast Press, Inc. p. 263-283.

8. Villa, P. and E. Mahieu, *Breakage patterns of human long bones.* Journal of Human Evolution, 1991. **21**: p. 27-48.

9. Lyman, R.L., *Vertebrate Taphonomy*. 1994, Cambridge: Cambridge University Press.

10. Galloway, A., *Fracture patterns and skeletal morphology: the lower extremity*, in *Broken Bones: Anthropological Analysis of Blunt Force Trauma*, A. Galloway, Editor. 1999, Charles C. Thomas: Springfield, IL. p. 160-223.

11. Galloway, A., *Fracture patterns and skeletal morphology: the upper extremity*, in *Broken Bones: Anthropological Analysis of Blunt Force Trauma*, A. Galloway, Editor. 1999, Charles C. Thomas: Springfield, IL. p. 113-159.

12. Galloway, A., *Fracture patterns and skeletal morphology: introduction and the skull*, in *Broken Bones: Anthropological Analysis of Blunt Force Trauma*, A. Galloway, Editor. 1999, Charles C. Thomas: Springfield, IL. p. 63-80.

13. Galloway, A., *The biomechanics of fracture production*, in *Broken Bones: Anthropological Analysis of Blunt Force Trauma*, A. Galloway, Editor. 1999, Charles C. Thomas: Springfield, IL. p. 35-62.

14. Marshall, L.G., *Bone modification and “ the laws of burial"*, in *Bone Modification*, R. Bonnischen and M.H. Sorg, Editors. 1989, University of Maine Center for the Study of the First Americans.: Orono. p. 7-24.

15. Behrensmeyer, A., *Taphonomic and ecologic information from bone weathering.* Paleobiology, 1978. **4**(2): p. 150-162.

16. Hill, A., *On carnivore and weathering damage to bone.* Current Anthropology, 1976. **17**: p. 335-336.

17. Lyman, R. and G. Fox, *A critical evaluation of bone weathering as an indication of bone assemblage formation*, in *Forensic taphonomy. The post-mortem fate of human remains*, W. Haglund and M. Sorg, Editors. 1997, CRC Press: Boca Raton. p. 223-247.

18. Lyman, R.L. and G.L. Fox, *A critical evaluation of bone weathering as an indication of bone assemblage formation.* Journal of Archaeological Science, 1989. **16**: p. 293-317.

19. Tappen, N., *The relationship of weathering cracks to split-line orientation in bone.* American Journal of Physical Anthropology, 1969. **31**(2): p. 191-197.

20. Tappen, N., *Advanced weathering cracks as an improvement on split-line orientation in bone.* American Journal of Physical Anthropology, 1976. **44**: p. 373-377.

21. Tappen, N. and R. Peske, *Weathering cracks and split-line patterns in archaeological bone.* American Antiquity, 1970. **35**: p. 383-386.

22. Pokines, J.T. and J.E. Baker, *Effects of burial environment on osseous remains*, in *Manual of Forensic Taphonomy*, J. Pokines and S. Symes, Editors. 2014, CRC Press: Boca Raton, FL. p. 73-114.

23. Crow, P., *Mineral weathering in forest soils and its relevance to the preservation of the buried archaeological resource.* Journal of Archaeological Science, 2008. **35**: p. 2262-2273.

24. Dirks, P.H.G.M., et al., *Geological and taphonomic context for the new hominin species Homo naledi from the Dinaledi Chamber, South Africa.* eLife, 2015. **4**: p. e09561.

25. Backwell, L.R., et al., *Criteria for identifying bone modification by termites in the fossil record.* Palaeogeography, Palaeoclimatology, Palaeoecology, 2012. **337-338**: p. 72-87.

26. Evans, T., *Fluvial taphonomy*, in *Manual of Forensic Taphonomy*, J.T. Pokines and S.A. Symes, Editors. 2014, CRC Press: Boca Raton, FL. p. 115-142.

27. Bassett, H.E. and M.H. Manhein, *Fluvial transport of human remains in the lower Mississippi River.* Journal of Forensic Sciences, 2002. **47**(4): p. 719-724.

28. Nawrocki, S.P., et al., *Fluvial Transport of Human Crania*, in *Forensic Taphonomy: The Postmortem Fate of Human Remains*, W.D. Haglund and M.H. Sorg, Editors. 1997, CRC Press: London. p. 529-552.

29. Behrensmeyer, A.K., *Time resolution in fluvial vertebrate assemblages.* Paleobiology, 1982. **8**: p. 211-227.

30. Behrensmeyer, A.K., *Vertebrate preservation in fluvial channels.* Palaeogeography, Palaeoclimatology, Palaeoecology, 1988. **63**: p. 183-199.

31. Hanson, C.B., *Fluvial taphonomic processes: models and experiments*, in *Fossils in the Making*, A.K. Behrensmeyer and A.P. Hill, Editors. 1980, Chicago University Press: Chicago. p. 156-181.

32. Parkinson, A.H., *Traces of insect activity at Cooper’s D fossil site (Cradle of Humankind, South Africa).* Ichnos, 2016.

33. Bartelink, E.J., J.M. Wiersema, and R.S. Demaree, *Quantitative analysis of sharp-force trauma: an application of scanning electron microscopy in forensic anthropology.* Journal of Forensic Sciences, 2001. **46**(6): p. 1288-1293.

34. Symes, S.A., et al., *Taphonomic context of sharp-force trauma in suspected cases of human mutilation and dismemberment*, in *Advances in Forensic Taphonomy: Method, Theory and Archeological Perspectives*, M.H. Sorg and W.D. Haglund, Editors. 2002, CRC Press: Boca Raton, FL. p. 403-434.

35. Alunni-Perret, V., et al., *Scanning electron microscopy analysis of experimental bone hacking trauma.* Journal of Forensic Sciences, 2005. **50**: p. 796-801.

36. Lynn, K.S. and S.I. Fairgrieve, *Macroscopic Analysis of Axe and Hatchet Trauma in Fleshed and Defleshed Mammalian Long Bones.* Journal of Forensic Sciences, 2009. **54**(4): p. 786-792.

37. Lynn, K.S. and S.I. Fairgrieve, *Microscopic indicators of axe and hatchet trauma in fleshed and defleshed mammalian long bones.* Journal of Forensic Sciences, 2009. **54**(4): p. 793-797.

38. Tucker, B.K., et al., *Microscopic and macroscopic characteristics of hacking trauma.* Journal of Forensic Science, 2001. **46**(2): p. 234-240.

39. Pante, M.C. and R.J. Blumenschine, *Fluvial transport of hominin-and carnivore-modified long bone fragments.* American Journal of Physical Anthropology, 2009: p. 205-205.

40. Pokines, J.T., *Faunal dispersal, reconcentration, and gnawing damage to bone in terrestrial environments*, in *Manual of Forensic Taphonomy*, J. Pokines and S. Symes, Editors. 2013, CRC Press: Boca Raton, FL. p. 201-248.

41. Symes, S.A., et al., *Patterned thermal destruction of human remains in a forensic setting*, in *The Analysis of Burned Human Remains*, C.W. Schmidt and S.A. Symes, Editors. 2008, Academic Press: London. p. 15-54.

42. Bristow, J., Z. Simms, and P.S. Randolph-Quinney, *Taphonomy*, in *Forensic Anthropology 2000-2010*, B. S. and E. Ferguson, Editors. 2011, CRC Press: Boca Raton, FL. p. 279-318.
